# Attenuated hypocholesterolemia following severe trauma signals risk for late ventilator-associated pneumonia, ventilator dependency, and death: a retrospective study of consecutive patients

**DOI:** 10.1186/1476-511X-10-42

**Published:** 2011-03-03

**Authors:** C Michael Dunham, Thomas J Chirichella

**Affiliations:** 1 Trauma/Critical Services, St. Elizabeth Health Center, 1044 Belmont Avenue, Youngstown, OH, 44501, USA

## Abstract

**Background:**

Post-traumatic ventilator-associated pneumonia (VAP) is a substantial clinical problem that increases hospital costs and typically adds to the duration of mechanical ventilation. We evaluated the impact of VAP on ventilator days. We also assessed 48-hour total blood cholesterol (TC) and other potential risk factors for the development of VAP.

**Methods:**

We performed a retrospective study of consecutive trauma patients requiring emergency tracheal intubation and evaluated TC, age, gender, ethanol status, smoker status, injury mechanism, chest injury, brain injury, Injury Severity Score (ISS), shock, day-one hypoxemia, and RBC transfusion as potential risks for VAP.

**Results:**

The 152 patients had ISS 28.1, brain injury 68.4%, VAP 50.0%, ventilator days 14.3, and death 9.9%. Ventilator days were increased with late VAP (p < 0.0001). TC was 110.7 mg/dL with expected TC 197.5 mg/dL. TC was lower with chest injury, shock, and RBC transfusion but, higher with brain injury (p ≤ 0.01). TC decreased as ISS increased (p = 0.01). However, one patient subset (ISS ≥ 20-&-TC ≥ 90 mg/dL) had a relative increase in TC despite an increase in ISS. ISS ≥ 20-&-TC ≥ 90 mg/dL, but not ISS alone, was the only independent predictor of late VAP (OR 3.0; p = 0.002). ISS ≥ 20-&-TC ≥ 90 mg/dL and day-one hypoxemia were the only independent predictors for increased ventilator days (p = 0.01). ISS ≥ 20-&-TC ≥ 90 mg/dL, but not ISS alone, was the only predictor of death (OR 3.8; p = 0.03).

**Conclusions:**

Severe traumatic injury produced substantial hypocholesterolemia that is greater with chest injury, shock, and RBC transfusion, but less with brain injury. Total blood cholesterol tended to decrease with increasing injury severity. However, attenuated hypocholesterolemia (ISS ≥ 20-&-TC ≥ 90 mg/dL) represents a unique response that can occur with critical injury. Attenuated hypocholesterolemia signals early risk for late VAP, ventilator dependency, and death.

## Background

Ventilator-associated pneumonia (VAP) rates following traumatic injury have been shown to be variable and range from less than 20% [1,2] to as high as 40-60% [2-5]. Of interest, some investigators have implied there is a virtual elimination of posttraumatic VAP with the implementation of ventilator bundle management [6]. However, other studies indicate that VAP rates with traumatic brain injury are substantial, 32-45% [4,5]. Due to postinjury pulmonary aspiration risk, VAP is likely to continue as a vexing problem for institutions managing patients with severe brain injury.

It is controversial whether VAP has an influence on death. Some investigators have found that VAP increases death, [2,5] while others did not. Studies showing no impact on death include those with [7] and without [4] control groups. Compellingly, numerous trauma patient studies have demonstrated that VAP, when compared to non-VAP patients, prolongs the duration of mechanical ventilation [4-7].

Investigators have shown that the inflammatory response at 48 hours postinjury indicates the risk for development of VAP. Woiciechowsky [8] showed that subsequent VAP development relates to higher levels of Interleukin-6, while Cohen [9] found a relationship with plasma Protein C values.

Because multiple studies have implied that emergency tracheal intubation in trauma patients increases VAP rates, [1,2,7] we undertook a retrospective review of consecutive trauma patients undergoing emergency tracheal intubation. The primary purpose of the study was to evaluate the impact of VAP on duration of mechanical ventilation. The secondary objective was to assess potential early risk factors, e.g., Injury Severity Score (ISS) and smoking status, for the development of VAP. Because the first author has published on total blood cholesterol (TC) in other trauma populations, [10,11] we monitor TC, an inflammatory marker, daily in our intensive care unit (ICU). Based on the work by Woiciechowsky and Cohen, we selected 48-hour TC as a potential risk factor for VAP. Since phenytoin can influence TC levels [12,13] and many patients undergoing emergency tracheal intubation receive phenytoin for severe brain injury, we evaluated the relationship between 48-hour TC and phenytoin loading.

## Methods

Study eligibility criteria were consecutive trauma patients requiring emergency tracheal intubation within 24 hours of injury and mechanical ventilation for greater than or equal two days. Early risk factor analysis for VAP included the following variables: age, gender, ethanol status, smoking status, mechanism of injury, Injury Severity Score (ISS), chest injury, brain injury, shock, red blood cell (RBC) transfusion, day-one hypoxemia, and 48-hour TC. A univariate statistical assessment was performed for each potential risk factor and VAP. 48-hour TC was then interacted with the other potential risk factors to determine if there is a statistical relationship with VAP. Information in the study database came from a Level I Trauma Registry and, as needed, medical record review. The Institutional Review Board waived the need for informed consent.

The following statements provide clarification of study methodology and variable definitions. Day-one hypoxemia indicates a PaO_2_/FiO_2 _< 200 during the first 24 hours postinjury. VAP criteria included: 1) the presence of two or more Systemic Inflammatory Response Syndrome (SIRS) criteria, 2) new or progressive radiographic pulmonary infiltrate, and 3) a laboratory report with a) moderate or abundant culture growth of a bacterial pathogen, or b) polymicrobial growth with moderate or abundant polymorphonuclear white blood cells on sputum gram stain. Sputum specimens were harvested using bronchoscopic-directed or blind-aspiration methods, without alveolar lavage. Using the Centers for Disease Control criteria, early VAP occurs in the first-48 hours postinjury, while late VAP takes place after the first-48 hours. Mean Expected TC values were from a large, United States, population-based survey, according to gender and age range. Because pre-injury TC values were not available, Expected TC served as a relative reference for the postinjury TC values. Sources for Expected TC emanated from the Centers for Disease Control and Prevention using 2003-2006 data (GOOGLE search: "Health, United States, 2009"; see table 69) and Arnett's study [14] using 2000-2002 data. Fractional TC is the 48-hour TC value divided by the Expected TC value. The TC Difference is the expected TC value minus the 48-hour TC value.

We identified which brain-injured patients underwent phenytoin loading on the day of injury. Phenytoin loading was documented when there is a written order and validation in the medication administration record. Phenytoin serum level results were assessed at 24-48 hours after loading.

We used the SAS System for Windows, release 9.2 (SAS Institute Inc., Cary, NC, USA) to perform statistical analysis. We expressed continuous variables as mean and standard deviation. We assessed statistical relationships using the following techniques: a) t-test for comparison of interval continuous data between two groups; b) Wilcoxon rank-sum test for comparison of ordinal-rank continuous data between two groups; c) Pearson correlation coefficient analysis to assess the relationship between two continuous variables; d) Fisher's exact test to assess 2 × 2 contingency tables; e) multivariate regression analysis to assess independent variable impact on continuous response variables; and f) logistic multivariate regression analysis to assess independent variable impact on binary response variables. We considered p < 0.05 to represent statistical significance.

## Results

From August 2006 to June 2008, 152 consecutive patients underwent 2,170 days of mechanical ventilation. Traits of the study patients and outcomes are in Table 1. Of the 152 patients, 76 (50.0%) had VAP (early and/or late). Early VAP without late VAP occurred in 19 patients, late VAP without early VAP occurred in 43 patients, and late VAP with early VAP occurred in 14 patients. The late VAP rate (without or with early VAP) was 37.5% (57/152). After excluding the seven patients dying in the first six days, the influence of VAP on duration of mechanical ventilation was determined. Of the 73 patients without VAP, the duration of ventilation was 11.1 ± 7.8 days. The 17 patients with early VAP, but no late VAP underwent 12.3 ± 9.0 days of ventilation (p = 0.54). The duration of mechanical ventilation for the 90 patients without late VAP was 11.4 ± 8.0 days, whereas the 55 patients with late VAP had 20.3 ± 12.3 ventilator days (p < 0.0001).

**Table 1:** Injury Characteristics and Outcomes of Emergently Intubated Patients

Characteristic	Data
Age (years)	42.5 ± 19
Male	113 (74.3%)
Ethanol Positive	48 (31.6%)
Smoker	53 (34.9%)
Blunt trauma	141 (92.8%)
ISS	28.1 ± 12.4
ISS ≥ 20	113 (74.3%)
Chest injury	72 (47.4%)
Head AIS	3.0 ± 2.0
Brain injury	104 (68.4%)
GCS	9.4 ± 4.5
GCS ≤ 8	74 (48.7%)
Shock	43 (28.3%)
RBC transfusion	53 (34.9%)
Day-one Hypoxemia	74 (48.7%)
VAP	76 (50.0%)
Expected TC (mg/dL)	197.5 ± 15.5
48-hour TC (mg/dL)	110.7 ± 30.3
Fractional TC	0.560 ± 0.15
TC Difference (mg/dL)	86.8 ± 29.5
Ventilator days	14.3 ± 10.7
ICU days	17.2 ± 10.5
Hospital LOS	20.4 ± 11.0
Death	15 (9.9%)

ISS, Injury Severity Score; AIS, Abbreviated Injury Scale; GCS, Glasgow Coma Score; RBC, red blood cell; VAP, ventilator-associated pneumonia; TC, total cholesterol; ICU, intensive care unit; LOS, length of stay

The risk factor analysis demonstrated that age, gender, ethanol status, smoker status, mechanism of injury, chest injury, brain injury, shock, and day-one hypoxemia rates and RBC transfusion amounts did not differ without or with late VAP (p >> 0.05). ISS values were higher in patients with late VAP (without or with early VAP) (see Table 2). The interaction between ISS and TC (ISS × TC) was higher with late VAP and created a more significant relationship with late VAP than did ISS alone (see Table 2). Using logistic regression analysis, late VAP had an association with ISS × TC (p = 0.03), but not ISS (p = 0.58). ISS was dichotomized into the following groups: ≥ 15, ≥ 20, ≥ 25, and ≥ 30. The ISS ≥ 20 dichotomy yielded the greatest accuracy and sensitivity for late VAP. The ISS and TC interaction was dichotomized to evaluate its relationship with late VAP (see Table 3). The ISS ≥ 20-&-TC ≥ 90 mg/dL dichotomy produced the best balance between accuracy and sensitivity with late VAP. The late VAP rate with ISS ≥ 20-&-TC ≥ 90 mg/dL was 48.8% (40/82) and in the group without ISS ≥ 20-&-TC ≥ 90 mg/dL was 24.3% (17/70). Using logistic regression analysis, late VAP had an association with ISS ≥ 20-&-TC ≥ 90 mg/dL (p = 0.009), but not ISS (p = 0.27).

**Table 2:** ISS ≥ 20 and TC ≥ 90 mg/dL is a Risk for Late VAP

	No LVAP	LVAP	P Value
**Patients**	95 (62.5%)	57 (37.5%)	
**48-hour TC **(mg/dL)	108.6 ± 31.6	114.3 ± 27.8	0.26
**ISS**	26.5 ± 11.9	30.6 ± 13.0	0.048
**ISS × TC**	2762.4 ± 1243.7	3487.6 ± 1789.2	0.004

ISS, Injury Severity Score; TC, total cholesterol; VAP, ventilator-associated pneumonia; LVAP, late VAP

**Table 3:** Dichotomized ISS × 48-hour TC Relationship with Late VAP

ISS	48-hr. TC	Accuracy	% of 152	LVAP Sensitivity	LVAP PPV	OR	P Value
**≥ 20**	**≥ 100 mg/dL**	61.8%	41.5%	54.4%	49.2%	2.4	0.01
**≥ 20**	**≥ 90 mg/dL**	61.2%	53.9%	70.2%	48.8%	3.0	0.002
**≥ 20**	**≥ 80 mg/dL**	57.9%	63.8%	79.0%	46.4%	3.1	0.003
**≥ 20**	**≥ 70 mg/dL**	53.9%	69.1%	80.7%	43.8%	2.6	0.02

ISS, Injury Severity Score; TC, total cholesterol; LVAP, late ventilatory-associated pneumonia; PPV, positive predictive value; OR, odds ratio.

Patients with chest injury, shock, or RBC transfusion had greater hypocholesterolemia (HC) than those without these conditions (see Table 4). Brain injury patients had less HC then those without brain injury (see Table 4). Of the 152 patients, 69 (45.4%) had phenytoin loading with a therapeutic level in 66 (95.6%) at 24-48 hours. TC levels were higher with phenytoin loading (115.7 ± 30.9) in comparison to those without loading (106.5 ± 29.3; p = 0.06). Multivariate regression analysis showed that TC levels relate to brain injury (p = 0.006), but not phenytoin loading (p = 0.92).

**Table 4:** 48-hour Total Cholesterol Levels by Injury Traits

	No Chest Injury	Chest Injury	P Value
**Number**	80 (52.6%)	72 (47.4%)	--
**TC **(mg/dL)	119.8 ± 28.5	100.6 ± 29.1	0.0001
**ISS**	24.3 ± 12.0	32.3 ± 11.5	0.0001
			
	**No Shock**	**Shock**	
**Number**	109 (71.7%)	43 (28.3%)	--
**TC **(mg/dL)	114.5 ± 31.1	101.0 ± 26.1	0.01
**ISS**	26.4 ± 11.5	32.2 ± 13.7	0.01
			
	**No Transfusion**	**Transfusion**	
**Number**	99 (65.1%)	53 (34.9%)	--
**TC **(mg/dL)	121.2 ± 29.5	91.2 ± 20.6	0.0001
**ISS**	25.1 ± 12.5	33.6 ± 10.2	0.0001
			
	**No Brain Injury**	**Brain Injury**	
**Number**	48 (31.6%)	104 (68.4%)	--
**TC **(mg/dL)	98.8 ± 27.7	116.2 ± 30.0	0.0008
**ISS**	24.5 ± 14.4	29.7 ± 11.1	0.02

TC, total cholesterol; ISS, Injury Severity Score

Of the 57 late VAP patients, the mean 48-hour TC was 114.3 ± 27.8. Subsequently, the late VAP TC mean decreased to 94.5 ± 25.8; p = 0.0002. At discharge from the ICU, the late VAP TC mean increased to 130.7 ± 45.1; p = 0.0001.

**Figure 1:**
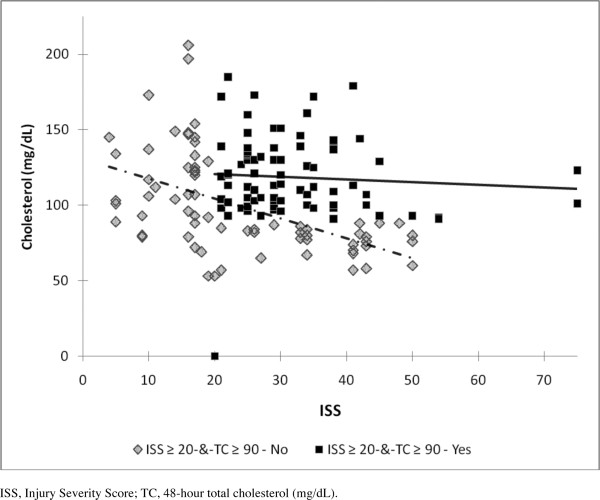
**The relationship between 48-hour total cholesterol and Injury Severity Score**. The ISS ≥ 20-&-TC ≥ 90 mg/dL - Yes group (boxes) shows no statistical relationship between cholesterol and Injury Severity Score (r = -0.07; p = 0.55). The ISS ≥ 20-&-TC ≥ 90 mg/dL - No group (diamonds) demonstrates an inverse correlation between cholesterol and Injury Severity Score (r = -0.53; p < 0.0001).

The ISS ≥ 20-&-TC ≥ 90 mg/dL group had a higher ISS and 48-hour TC than the other group; see Table 5. For the 152 patients, TC had a weak, inverse relationship with ISS (r = -0.20; p = 0.01). With ISS ≥ 30, TC was lower (102.9 ± 28.6 mg/dL) when compared to ISS < 30 (115.9 ± 30.4 mg/dL; p = 0.009). The ISS ≥ 20-&-TC ≥ 90 mg/dL group had no significant relationship between ISS and TC (n = 82; r = -0.07; p = 0.55). See Figure 1 However, the other group had a significant inverse association between ISS and TC (n = 70; r = -0.53; p < 0.0001).

**Table 5:** ISS ≥ 20 and TC ≥ 90 mg/dL Traits

ISS ≥ 20-&-TC ≥ 90 mg/dL	No	Yes	P Value
**Number**	70	82	--
**48-hour TC **(mg/dL)	99.3 ± 33.2	120.5 ± 23.6	0.0001
**ISS**	23.9 ± 13.3	31.7 ± 10.4	0.0001
**ISS × TC**	2140.4 ± 950.4	3797.5 ± 1479.8	0.0001

ISS, Injury Severity Score; TC, total cholesterol; RBC, red blood cell

The mechanism of injury, chest injury, brain injury, shock, day-one hypoxemia, VAP, late VAP rates, RBC transfusion, ISS, and 48-hour TC did not differ in 137 surviving and 15 dying patients (p >> 0.05). However, ISS × TC was higher in the deaths (3828.3 ± 1495.3) when compared to the survivors (2947.4 ± 1489.2; p = 0.03). The ISS ≥ 20-&-TC ≥ 90 mg/dL rate was also higher in those dying (80.0% [70/137]) when compared to those living (51.1% [12/15]; OR = 3.8; p = 0.03). The death rate with ISS ≥ 20-&-TC ≥ 90 mg/dL was 14.6% (12/82) and the rate without ISS ≥ 20-&-TC ≥ 90 mg/dL was 4.3% (3/70). The death and ISS ≥ 20-&-TC ≥ 90 mg/dL relationship had a sensitivity of 80.0%, positive predictive value of 14.6%, negative predictive value of 95.7%, odds ratio of 3.8, and p = 0.03.

Further analysis excluded the seven patients dying during the first 6 days. The remaining 145 patients accounted for 95.4% of the patients. Of the 145 patients, 75 (51.7%) had ISS ≥ 20-&-TC ≥ 90 mg/dL and 70 (48.3%) did not. Late VAP occurred in 55 (37.9%). The potential early predictors, age, gender, ethanol status, smoker status, mechanism of injury, chest injury, brain injury, and shock rates, RBC transfusion amounts, and 48-hour TC values did not influence the duration of mechanical ventilation (p >> 0.05). The duration of mechanical ventilation increased with ISS (p = 0.03), day-one hypoxemia (p = 0.01), ISS × TC (p = 0.048), and ISS ≥ 20-&-TC ≥ 90 mg/dL (p = 0.02). Using multivariate regression analysis, duration of mechanical ventilation had an association with day-one hypoxemia (p = 0.01) and ISS ≥ 20-&-TC ≥ 90 mg/dL (p = 0.01), but not ISS (p = 0.19).

## Discussion

The study group undergoing emergency tracheal intubation principally had blunt trauma, serious anatomic injuries (according to ISS), and severe brain injury. Outcomes included a VAP rate of 50%, two weeks of mechanical ventilation, and a 10% death rate. Substantial HC occurred at 48 hours postinjury and late VAP had an association with increased days of mechanical ventilation. Other investigators have also found significant HC following traumatic injury [10,11,15-17] and an increase in ventilator days with VAP [4-7]. Of the numerous potential risk factors assessed, only the interactive variable, ISS ≥ 20-&-TC ≥ 90 mg/dL, had an independent association with late VAP. ISS ≥ 20-&-TC ≥ 90 mg/dL also had a significant relationship with increased mortality and duration of mechanical ventilation.

### ISS ≥ 20-&-TC ≥ 90 mg/dL is a risk for late VAP, death, and ventilator dependency

We found very few early, postinjury predictors for the subsequent development of late VAP. Specifically, age, gender, ethanol, smoker, mechanism of injury, chest injury, brain injury, shock, RBC transfusion, and day-one hypoxemia status were not risk factors for late VAP (without or with early VAP). On the other hand, ISS, ISS × TC, and ISS ≥ 20-&-TC ≥ 90 mg/dL were higher in patients with late VAP. Of great interest, ISS × TC and ISS ≥ 20-&-TC ≥ 90 mg/dL were independent predictors of late VAP, whereas ISS was not. Thus, the interaction of ISS and TC had a stronger association with late VAP than did ISS alone. Accordingly, ISS ≥ 20-&-TC ≥ 90 mg/dL signals an early jeopardy for late VAP and its association with greater ventilator dependency.

Almost all potential early risk factors failed to differ in surviving and dying patients. These variables included blunt trauma mechanism, chest injury, brain injury, shock, RBC transfusion, day-one hypoxemia, VAP, late VAP, 48-hour TC, and ISS. However, the ISS-TC interaction variables (ISS × TC and ISS ≥ 20-&-TC ≥ 90 mg/dL) were significantly greater for nonsurvivors.

Similar to the findings with late VAP and death, most potential early risk factors failed to have an association with mechanical ventilation duration. However, the duration of mechanical ventilation increased with ISS, day-one hypoxemia, ISS × TC, and ISS ≥ 20-&-TC ≥ 90 mg/dL. Using multivariate regression analysis, ISS ≥ 20-&-TC ≥ 90 mg/dL and day-one hypoxemia had an independent significant relationship with ventilator days. However, ISS did not have an independent effect with day-one hypoxemia status. The multiple relationships of ISS ≥ 20-&-TC ≥ 90 mg/dL with late VAP, death, and days of mechanical ventilation imply that the interaction of ISS with 48-hour TC has a greater association than ISS alone.

### Factors influencing hypocholesterolemia

A comparison of the 48-hour TC values with the expected levels (Fractional TC and TC Difference), from a large population-based survey, provides a perspective for the magnitude of HC. This evaluation showed a reduction of 48-hour postinjury TC to be almost 50% of the expected levels, with a decrement in TC near 90 mg/dL. Others have also found substantial HC following traumatic injury [10,11,15-17]. Several injury traits had an association with the magnitude of 48-hour postinjury HC. Our study shows that as ISS increased there was a tendency for greater HC. Although this was statistically significant, it is clear there was individual variability. In particular, it is important to note that the ISS ≥ 20-&-TC ≥ 90 mg/dL demonstrated no variation of TC with ISS. However, the other group showed a highly significant inverse relationship between TC and ISS. Patients with chest injury, shock, or those requiring RBC transfusion had greater HC than those without these conditions. Although patients with brain injury had HC, it was less severe than for those without brain injury. We are not aware of any study that describes the influence of these traits on TC levels in trauma patients. In a prospective study of critically ill surgical patients, Gordon [18] found that patients with neurologic conditions had markedly less HC in comparison to those without brain pathology. The Gordon manuscript indicates that most patients with neurologic surgical conditions were receiving phenytoin, which may account for the diminution of HC. Investigations have demonstrated that phenytoin can increase TC values; [12,13] however, the study time-frame was typically several weeks [12]. Phenytoin is an inducer of the cytochrome P450 enzyme system [12] and this enzymatic milieu plays an important role in cholesterol balance [19]. Opinions in the literature suggest that cytochrome P450 induction is not likely for several days or weeks and is, in part, related to a drug's half-life [13,20]. Further, acute inflammation (release of cytokines) likely inhibits cytochrome P450 metabolism [20]. Based on the short time from phenytoin loading until obtaining the 48-hour TC value, cytochrome P450 induction seems improbable. Additionally, the fact this occurs during acute, severe injury with systemic inflammation also decreases the likelihood of cytochrome P450 induction. The multivariate analysis of phenytoin and brain injury effect on 48-hour TC strongly indicates that phenytoin did not influence TC values.

For patients developing late VAP, we reviewed all TC values throughout their ICU course. Following the 48-hour postinjury TC assessment, patients with late VAP had a subsequent further decrease in TC. This is consistent with our previous documentation [11] and that of other investigators who find HC with infectious complications [10,16,21,22]. After this TC nadir, resolution of late VAP showed an improvement in HC with a significant increase in TC. Again, we previously demonstrated this proclivity toward resolution of HC with clinical stabilization [11]. Alvarez [23] and Stachon [24] also provide evidence that HC improves with patient stabilization following infection and major surgery.

### HC is typical following traumatic, surgical, and infectious injury

After combining and critically reviewing an expanding literature, there is an impression that HC is the conventional, if not universal, human response to severe trauma, [10,11,15-17] surgery, [18,24-26] infection, [10,11,16,21,22] and critical illness [27]. It is also noteworthy the degree of HC has been shown to correlate with death in ICU, [27] general surgical, [25,26] trauma, [16] and cardiothoracic surgical [24] patients. It is also compelling that patients with resolution of infection and discharged alive from the ICU have been shown to have a significant improvement in HC [11,24]. However, dying patients were found to have persistent HC [11,24]. There is growing evidence that HC is a manifestation of systemic inflammatory up-regulation. Apropos, studies have provided data documenting a relationship between HC and systemic inflammatory mediators and markers [18,21,24].

### Postinjury inflammation may be adaptive or maladaptive

Substantial evidence exists that major injury leads to systemic inflammatory up-regulation [28-30]. Following postinjury systemic inflammatory up-regulation, an anti-inflammatory response typically ensues [28,31]. Such postinjury systemic inflammatory responses are typically adaptive [29,30]. However, the response may be maladaptive, leading to organ failure, infection, and death [7-9,29].

Literature evidence suggests that the degree of HC and systemic inflammation tends to increase with the severity of traumatic injury [31-33] and surgical illness [18,34]. The current study demonstrates a significant, but weak, inverse relationship between TC and ISS and a relatively large TC standard deviation with ISS ≥ 30 patients. These findings indicate that TC may not routinely decrease with greater injury severity. An attenuation of postinjury HC, relative to an increasing ISS, appears to be a maladaptive, unexpected host response. The increase in late VAP, death, and duration of mechanical ventilation with ISS ≥ 20-&-TC ≥ 90 mg/dL supports this notion.

### Study limitations

Several methodological limitations need consideration. Although this is a retrospective study, this is an analysis of consecutive trauma patients requiring emergency tracheal intubation and we consider the trauma registry to be a reliable database. However, data accuracy and quality from a retrospective, database source are recognized to be lower when compared to a prospective, dedicated database. Debate regarding the diagnosis of VAP is controversial. Some investigators have found similarities between clinical and bronchoalveolar lavage criteria [35-37]. Whereas, others believe quantitative lavage is the superior technique [38]. It is noteworthy that several other trauma investigators found a VAP rate similar to that of the current study [2-5]. Of importance, by using our clinical VAP definition, we portend a subset of patients with substantially greater ventilator dependency, a finding noted by several other investigators. We did not document patients with a pre-injury diagnosis of hypercholesterolemia and/or receiving cholesterol-lowering medications that might influence 48-hour TC. The lower TC values for patients with RBC transfusion suggest that resuscitation and plasma volume expansion (dilution) may have influenced TC levels. The failure to assess the impact of initial fluid volumes on 48-hour TC is a study weakness.

## Conclusions

The current study describes consecutive, trauma patients needing emergency tracheal intubation who had severe injury and compelling, subsequent hypocholesterolemia. Patients with chest injury, shock, and RBC transfusion had a greater degree of hypocholesterolemia than those without these entities. On the other hand, brain injury, which also produced hypocholesterolemia, had a relative increase in TC in comparison to those without brain injury. We found that late VAP substantially increased duration of mechanical ventilation. Of the numerous potential early risk factors for adverse outcomes (late VAP, death, and duration of mechanical ventilation), only ISS ≥ 20-&-TC ≥ 90 mg/dL consistently had an association. The lack of an association of the many early risk factors with adverse outcomes, yet the finding that ISS ≥ 20-&-TC ≥ 90 mg/dL had significant independent correlations, suggests this is not a fortuitous finding. ISS ≥ 20-&-TC ≥ 90 mg/dL implies that patients with this characteristic have an increasing ISS and a disproportionate increase in TC. In other words, these patients appear to have an attenuated postinjury hypocholesterolemia response. The above observations imply that attenuation of hypocholesterolemia after severe injury may be a maladaptive host response and signals risk for subsequent late VAP, ventilator dependency, and death.

## Competing interests

The authors declare that they have no competing interests.

## Authors' contributions

CMD conceived and designed the study. CMD reviewed medical records to document the presence of early or late VAP, 48-hour cholesterol values, and phenytoin loading. CMD performed data analysis and interpretation and contributed to critical manuscript revisions. TJC performed data analysis and interpretation, wrote the first draft, and contributed to critical manuscript revisions. Both authors read and approved the final manuscript.
